# Dynamics of Pulmonary Perfusion and Function Following Radical Treatment for Lung Tumors: A Prospective Comparative Study of Surgery, Radiotherapy, and Thermal Ablation

**DOI:** 10.3390/cancers18081213

**Published:** 2026-04-10

**Authors:** Aurimas Mačionis, Ieva Balčiūnaitė, Grytė Galnaitienė, Rūta Dubeikaitė, Gertrūda Maziliauskienė, Ieva Dimienė, Irena Nedzelskienė, Edita Mišeikytė-Kaubrienė, Lina Padervinskienė, Skaidrius Miliauskas, Donatas Vajauskas, Marius Žemaitis

**Affiliations:** 1 Department of Radiology, Medical Academy, Lithuanian University of Health Sciences, LT-44307 Kaunas, Lithuania; ieva.balciunaite@lsmu.lt (I.B.); gryte.galnaitiene@lsmu.lt (G.G.); ruta.dubeikaite@lsmu.lt (R.D.); gertruda.maziliauskiene@kaunoklinikos.lt (G.M.); lina.padervinskiene@lsmu.lt (L.P.); donatas.vajauskas@lsmu.lt (D.V.); 2 Department of Pulmonology, Medical Academy, Lithuanian University of Health Sciences, LT-44307 Kaunas, Lithuania; ieva.dimiene@lsmu.lt (I.D.); skaidrius.miliauskas@lsmu.lt (S.M.); marius.zemaitis@lsmu.lt (M.Ž.); 3 Department of Dental and Oral Diseases, Hospital of Lithuanian University of Health Sciences Kauno Klinikos, LT-50161 Kaunas, Lithuania; irena.nedzelskiene@lsmu.lt; 4 Department of Radiology, Faculty of Medicine, Vilnius University, LT-03101 Vilnius, Lithuania; edita.miseikyte@mf.vu.lt

**Keywords:** lung cancer, pulmonary perfusion, SPECT/CT, thermal ablation, radiotherapy, surgical resection, lung function, perfusion defects

## Abstract

**Background/Objectives**: Accurate assessment of pulmonary function is essential before planning radical lung cancer treatment. While spirometry reflects global lung function, perfusion imaging provides detailed information on regional perfusion patterns. This study aimed to characterize the pre-treatment profile of patients and compare the impact of surgical resection, radiotherapy, and thermal ablation on global pulmonary function and regional perfusion using SPECT/CT. **Methods**: In this prospective study of 68 patients, pre- and post-treatment assessments were conducted using lung perfusion SPECT/CT. While the entire cohort underwent imaging, longitudinal global pulmonary function (spirometry and gas diffusion) was analyzed for 45 patients who completed the three-month follow-up. Quantitative analysis included perfusion percentages and lung volumes, while a semi-quantitative scoring system evaluated the severity of perfusion defects. **Results**: In the overall cohort, the affected lung perfusion and volume significantly decreased (*p* = 0.002). Subgroup analysis revealed that the surgical resection group experienced significant reductions in perfusion (from 54.0% to 41.0%, *p* = 0.002) and volume (*p* < 0.001) of the affected lung, whereas no statistically significant changes were observed in the thermal ablation and radiotherapy groups (*p* > 0.05). Notably, 60.3% of patients presented with perfusion defects before treatment. Post-treatment spirometry parameters, particularly FEV1% (threshold 83.5%, AUC = 0.783), served as reliable predictors of persistent perfusion impairment. **Conclusions**: Radiotherapy and thermal ablation are lung-perfusion-sparing treatments compared to surgical resection. The high prevalence of pre-existing perfusion defects emphasizes the importance of incorporating lung perfusion SPECT/CT into routine pre-treatment evaluation to optimize treatment selection.

## Simple Summary

Determining the most suitable treatment for lung tumors requires a precise evaluation of how different therapies affect a patient’s lung function and perfusion. This study aimed to compare how surgery, radiotherapy, and thermal ablation impact overall lung function and regional perfusion using advanced medical imaging. Our findings show that while surgical resection leads to a significant loss of lung volume and perfusion in the treated area, radiotherapy and thermal ablation are much more effective at preserving healthy lung tissue. Additionally, we discovered that more than half of the patients had underlying perfusion defects even before treatment began. These results suggest that incorporating detailed perfusion imaging into routine assessments can help doctors choose safer treatment strategies, potentially improving the management and recovery of lung cancer patients.

## 1. Introduction

Accurate assessment of pulmonary function is essential before planning radical lung cancer treatment. According to the European Respiratory Society (ERS) and the European Society of Thoracic Surgeons (ESTS) guidelines, complete spirometry and diffusing lung capacity for carbon monoxide (DLCOc) testing are recommended for all surgical candidates, while perfusion imaging is generally reserved for patients with impaired ventilatory parameters [1]. However, spirometry reflects global lung function, whereas pulmonary perfusion imaging provides information on regional patterns of pulmonary blood flow [2]. Therefore, a combined approach may offer a more comprehensive understanding of lung function and could influence treatment selection.

According to the current guidelines of the European Association of Nuclear Medicine (EANM), hybrid imaging using single-photon emission computed tomography combined with computed tomography (SPECT/CT) is the preferred method for lung perfusion assessment [3]. Historically, pulmonary perfusion was evaluated using planar scintigraphy imaging, which does not accurately reflect the segmental or lobar anatomy of the lungs. The overlap of signals between anatomical regions in planar imaging may obscure subtle regional abnormalities. SPECT/CT technique enables precise localization of perfusion defects and allows correlation with anatomical alterations, such as tumor involvement, atelectasis or treatment-related fibrosis [4]. Despite these advantages, it is rarely used to evaluate the outcomes of radical treatment of lung tumors.

Radical lung therapies such as surgical resection, stereotactic radiotherapy and thermal ablation show significant differences in their invasiveness and potential impact on surrounding lung tissue [5,6]. However, limited data exist on how these modalities affect pulmonary function. There is a lack of studies evaluating changes in both global function and regional perfusion, highlighting the novelty and clinical relevance of this study.

Therefore, the aim of this study was to characterize the pre-treatment profile of patients undergoing radical treatment for lung tumors and to assess the impact of surgery, stereotactic radiotherapy and thermal ablation on global pulmonary function and regional perfusion using pre- and post-treatment lung function tests and perfusion SPECT/CT data.

## 2. Materials and Methods

### 2.1. Study Population

This was a single-center prospective, non-randomized comparative study conducted between 2022 and 2024 at the Hospital of Lithuanian University of Health Sciences. The study was approved by the Kaunas Regional Biomedical Research Ethics Committee in November 2022. This study was registered at ClinicalTrials.gov (identifier: NCT07272356).

Inclusion criteria were systemic therapy-naive NSCLC (stages Tis—T2) with no evidence of nodal involvement or distant metastasis or oligometastatic lung disease with ≤5 lesions, all considered suitable for surgery (SR), thermal ablation (TA), or stereotactic body radiotherapy (SBRT). The surgical group consisted of patients who underwent lobectomy, segmentectomy, or atypical resection. All participants had no significant comorbidities, physical or social limitations that could prevent participation in the study, no contraindications to general anesthesia, temporary cessation of antiplatelet therapy, or signs of severe coagulopathy. Requirements also included a documented multidisciplinary team recommendation for curative treatment (involving a pulmonologist, interventional radiologist, radiation oncologist, thoracic surgeon, and medical oncologist) and the patient’s signed informed consent for the planned therapy and participation in the study. This multidisciplinary approach was strictly followed to minimize selection bias, ensuring that treatment allocation was based on clinical consensus rather than individual provider preference. A detailed description of patient inclusion criteria, treatment allocation, and baseline demographic and histological characteristics of this cohort has been published previously [7].

All patients underwent lung perfusion scintigraphy SPECT/CT (single photon emission tomography with computed tomography) and lung function tests (spirometry and gas diffusion) prior to treatment. Follow-up testing was performed at 3 months (±2 weeks) after treatment. To address missing data for post-treatment lung function tests, a complete-case analysis was employed. Only patients who completed both pre- and post-treatment assessments were included in the longitudinal analysis of FEV1, FVC, and DLCOc.

### 2.2. Study Endpoints

The primary endpoint of this study was the change in regional pulmonary perfusion (measured in percentage, %) and regional lung volume (measured in milliliters, mL) of the treated lung at 3 months post-intervention compared to baseline, as assessed by SPECT/CT. Secondary endpoints included changes in global lung function parameters, specifically forced expiratory volume in 1 s (FEV1), forced vital capacity (FVC), and diffusing capacity of the lungs for carbon monoxide (DLCOc). Additionally, the correlation between pre-treatment perfusion defects and post-treatment functional outcomes was analyzed. This study is reported in accordance with the Strengthening the Reporting of Observational Studies in Epidemiology (STROBE) guidelines.

### 2.3. Lung Perfusion Scintigraphy

#### 2.3.1. Imaging Acquisition

Lung perfusion scintigraphy SPECT/CT was performed within 2 min after intravenous administration of 170 MBq (±21) of ^99m^Tc-labeled macroaggregated albumin (MAA; MAKRO-ALBUMON, Medi-Radiopharma Ltd., Érd, Hungary) in a fixed volume of 0.6 mL. During the tracer injection, the patient was in a supine position, and the MAA was administered via a slow injection spanning 3–4 breathing cycles to ensure optimal pulmonary distribution. The total number of administered MAA particles ranged from 200,000 to 400,000. To exclude injection infiltration or the formation of radioactive “hot spots,” all injections were performed through a safely placed intravenous catheter, which was flushed with saline prior to the ^99m^Tc-MAA administration.

SPECT/CT acquisitions were performed on a Veriton-CT solid-state, ring-shaped hybrid SPECT/CT scanner equipped with Cadmium Zinc Telluride (CZT) detectors (Spectrum Dynamics Medical Ltd., Caesarea, Israel). The protocol consisted of a five-minute SPECT acquisition using standard reconstruction parameters (Spectrum Dynamics), followed by low-dose CT (120 kV, 30 mA), with reconstructions of 2.5 mm for soft tissue and 1.25 mm for lung parenchyma. SPECT reconstruction was performed with a proprietary iterative algorithm (OSEM with resolution recovery), incorporating CT-based attenuation correction, scatter correction and Point Spread Function Recovery (PSFR). Image post-processing included a 3 × 3 × 3 voxel median smoothing filter. The CT-based attenuation maps were derived from the low-dose multi-slice CT component of the Veriton CT system (Spectrum Dynamics Medical). Reconstruction and processing were executed on the integrated operator console (Spectrum Dynamics). Hybrid SPECT/CT images were reconstructed into a multiplanar images format.

#### 2.3.2. Quantitative Analysis

Pulmonary perfusion SPECT/CT data were analyzed by an experienced nuclear medicine physician using MIM Software (version 6.9; MIMq Software Inc., Cleveland, OH, USA) with the lung lobar quantification module (Lung SPECT/CT Quant SD). Volumes of interest (VOIs) were automatically generated after manual delineation of pulmonary lobe fissures on low-dose CT data by the same observer.

The lungs were segmented on low-dose CT data into the right and left lungs. The VOIs were then applied to the hybrid SPECT/CT data. Lung perfusion percentages were calculated from SPECT counts:

Total counts in a lobe/total counts in both lungs × 100.

Lung volumes were derived from the CT data and expressed in milliliters (mL).

#### 2.3.3. Visual Analysis and Evaluation Criteria

Perfusion defects identified via scintigraphy that could not be attributed to underlying structural pathology on chest CT—such as tumor mass, atelectasis, or parenchymal disease—were included in the analysis.

Evaluation of lung perfusion defects is traditionally based on the PIOPED criteria to estimate the probability of PE: low or high likelihood. According to the 2019 EANM guidelines, interpretation of lung perfusion defects is simplified into two categories: PE present and PE absent [3].

In our study, we aimed to provide a more detailed assessment by considering both the presence and the extent or severity of perfusion defects. As ventilation imaging and CTPA were not part of the primary study protocol, we could not definitively confirm or rule out a diagnosis of pulmonary embolism (PE). Therefore, we developed a semi-quantitative scoring system for perfusion defects (Table 1). This scoring system was used by the experienced nuclear medicine physician to evaluate the pulmonary perfusion SPECT/CT data.

### 2.4. Lung Function Test

Forced expiratory volume in 1 s (FEV1) and forced vital capacity (FVC) were measured with a Ganshorn spirometry device (Ganshorn Medizin Electronic, Niederlauer, Germany). The values are described as L or % (predicted). FEV1 and the FEV1/FVC ratio are expressed in %. Gas diffusion was performed in a Ganshorn PowerCube Diffusion+ system (Ganshorn Medizin Electronic, Niederlauer, Germany). Diffusing capacity of the lungs adjusted for hemoglobin level (DLCOc) was assessed. The values are described as mmol/min/kPa or % (predicted). Pulmonary function tests were performed and interpreted according to European Respiratory Society (ERS) and American Thoracic Society (ATS) technical standards on interpretive strategies for routine lung function tests [8].

### 2.5. Statistical Analysis

Statistical data analysis was performed using the IBM SPSS Statistics 29.0 software package (IBM Corp., Armonk, NY, USA). Continuous variables are presented as means ± standard deviation (SD) or medians with interquartile ranges (IQRs), depending on the normality of the data distribution, which was assessed using the Shapiro–Wilk test. Categorical data were compared using the Chi-square (χ^2^) test; for small sample sizes, Fisher’s exact test or Monte Carlo methods were applied. To minimize selection bias, baseline characteristics were compared across groups using the Kruskal–Wallis test. Changes in global lung function parameters (FEV1, FVC, DLCOc) and regional perfusion parameters (perfusion share in % and volume) from baseline to follow-up were evaluated using the Wilcoxon signed-rank test. Differences between the three treatment modalities (surgical resection, thermal ablation, and stereotactic body radiotherapy) were determined using the Kruskal–Wallis test, with Bonferroni correction applied for post hoc pairwise comparisons. The diagnostic value of spirometry parameters in predicting regional perfusion defects was evaluated using Receiver Operating Characteristic (ROC) curve analysis, determining the Area Under the Curve (AUC) with 95% confidence intervals (CIs). Associations between established threshold values and perfusion impairments were expressed as odds ratios (ORs) with 95% CI. Perfusion defect severity over time was assessed by calculating the Spearman correlation coefficient (r). Results were considered statistically significant at *p* < 0.05.

## 3. Results

### 3.1. Descriptive Statistics

A total of 68 patients (39 males; 29 females; mean age, 70.2 ± 8.9 years; range, 46–91 years) who underwent lung perfusion imaging both before and after treatment were included in the study (Table 2).

**Figure 1:**
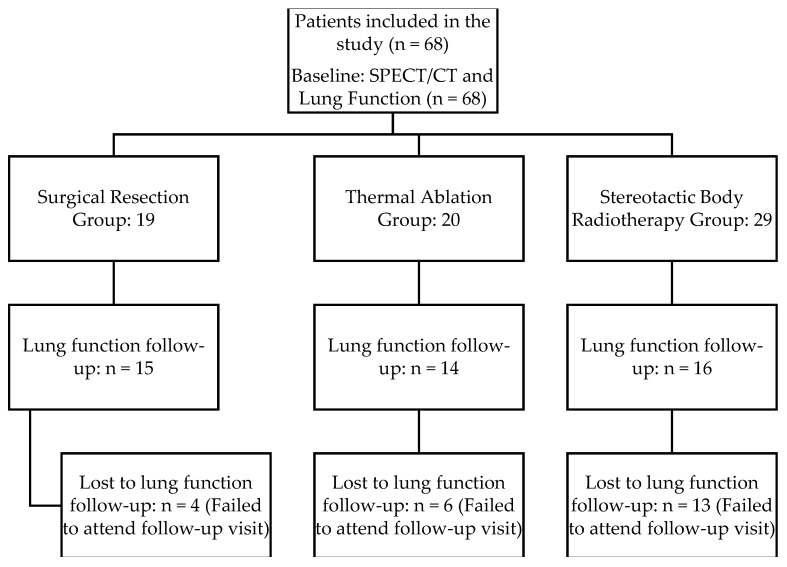
Participant flow diagram. While all 68 patients completed the post-treatment perfusion SPECT/CT imaging, follow-up lung function testing (spirometry and DLCOc) was available for 45 patients. The primary reason for missing follow-up data was the inability to attend the 3-month clinical visit.

However, the longitudinal analysis of pulmonary function (spirometry and DLCOc) was limited to the 45 patients who completed both baseline and 3-month follow-up assessments as 23 patients did not attend the follow-up testing due to physical limitations or non-attendance (Figure 1).

Of the total 68 patients included in the study, 29 (40.9%) underwent stereotactic body radiotherapy, 20 (30.3%) received thermal ablation, and 19 (28.8%) underwent surgical lung resection. The distribution of treatment types was not significantly different between the treatment groups (χ^2^ (2) = 0.76, *p* = 0.684). The right lung was affected in 42 patients (61.8%), the left lung in 25 (36.8%), and bilateral involvement was observed in one patient (1.4%), with no significant difference in the distribution of the affected lung side across treatment groups (χ^2^ (2) = 0.29, *p* = 0.290). Statistical analysis confirmed that baseline characteristics, such as age and lung side involvement, showed no significant differences between groups (*p* > 0.05), suggesting that the groups were comparable at the start of the study.

### 3.2. Lung Perfusion

#### 3.2.1. Lung Perfusion Percentages and Volume (Quantitative Parameters)

**Figure 2:**
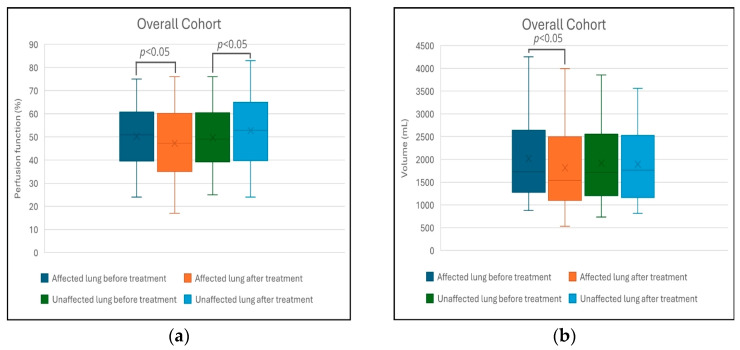

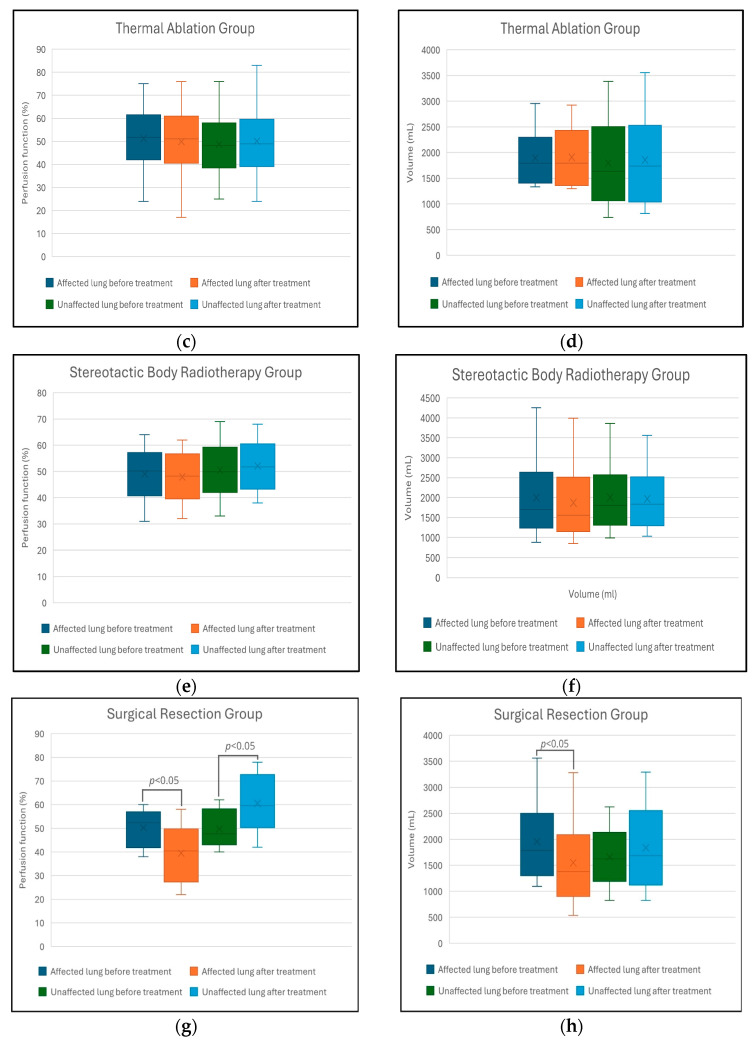
Comparison of Lung Perfusion and Volume Changes in Affected vs. Unaffected Lungs Across Different Treatment Modalities: (**a**) perfusion function (%) and (**b**) lung volume (mL) in the overall cohort; (**c**) perfusion function (%) and (**d**) lung volume (mL) in the thermal ablation group; (**e**) perfusion function (%) and (**f**) lung volume (mL) in the stereotactic body radiotherapy group; (**g**) perfusion function (%) and (**h**) lung volume (mL) in the surgical resection group. In each boxplot, the cross (×) indicates the mean and the horizontal line inside the box indicates the median. Statistical significance was defined as *p* < 0.05.

The changes in lung perfusion function (%) and lung volume (mL) in the overall cohort and across different treatment subgroups before and after treatment are summarized in Figure 2. In the overall cohort, perfusion of the affected lung significantly decreased from 51.5% (44.8–56.0) to 48.0% (45.0–55.0) (*p* = 0.002), while its volume decreased from 1635 mL (1414–2105) to 1413 mL (1286–2000) (*p* = 0.002). Additionally, perfusion of the unaffected lung increased from 48.5% (44.0–55.3) to 52.0% (45.0–59.0) (*p* < 0.001), while its volume showed no statistically significant change (*p* > 0.05).

Subgroup analysis showed no statistically significant changes in either perfusion or volume for patients treated with thermal ablation (*p* > 0.05) and radiotherapy (*p* > 0.05). In contrast, the surgical resection group demonstrated significant reductions in perfusion of the affected lung, which dropped from 54.0% (43.0–56.0) to 41.0% (29.0–47.0) (*p* = 0.002), while its volume decreased from 1750 mL (1370–2140) to 1303 mL (1026–1689) (*p* < 0.001). Furthermore, the unaffected lung showed a significant increase in perfusion from 46.0% (44.0–57.0) to 59.0% (53.0–71.0) (*p* = 0.002), but no statistically significant change in volume (*p* > 0.05).

Comparative analysis across treatment modalities demonstrated that surgical resection resulted in significantly greater changes in lung perfusion and volume compared to thermal ablation or radiotherapy (Table 3). Specifically, the resection group experienced a more pronounced decline in perfusion function within the affected lung (median 15.0%, IQR 1.0–19.0%), which was accompanied by a compensatory increase in perfusion of the unaffected lung. Furthermore, surgical intervention resulted in a substantial reduction in the volume of the affected lung (median 583 mL, IQR 223–637 mL). In contrast, changes in the volume of the unaffected lung did not differ significantly between the three treatment groups (*p* > 0.05).

#### 3.2.2. Lung Perfusion Defects (Qualitative Parameters)

**Figure 3:**
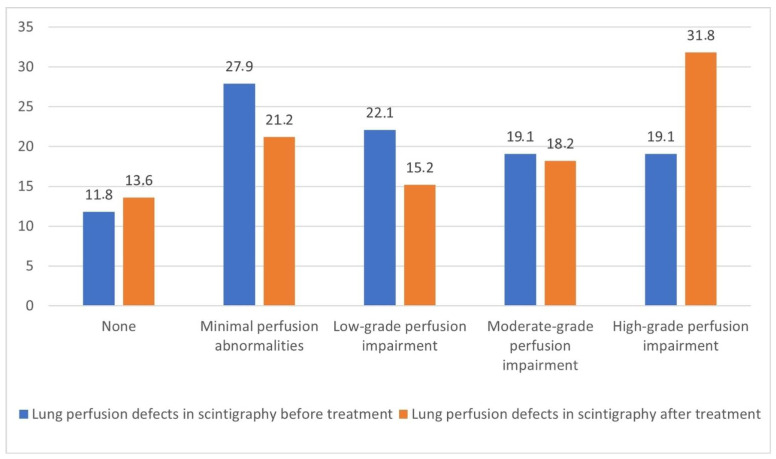
Percentage distribution of lung perfusion defects in scintigraphy before and after treatment (*n* = 68).

The distribution of pre-treatment lung perfusion defects, as shown in Figure 3, varied significantly across the groups. Perfusion defects were identified in 41 patients (60.3%) overall, with the highest prevalence observed in the thermal ablation (16/20, 80.0%) and stereotactic body radiotherapy (19/29, 65.5%) groups, compared to only 31.6% (6/19) in the surgical resection group.

Perfusion defects before treatment (χ^2^ = 11.471, df = 8, *p* = 0.175) and after treatment (χ^2^ = 7.911, df = 8, *p* = 0.460) did not differ significantly according to treatment type. No significant within-group changes in perfusion defect occurrence were found following treatment (*p* > 0.05). Perfusion defect severity remained highly consistent over time, as indicated by a significant correlation between pre- and post-treatment scores (r = 0.410, *p* < 0.001).

Perfusion defects in the entire cohort (Z = 1.563, *p* = 0.118) and in subgroups by treatment type (TA: Z = 0.361, *p* = 0.718; SBRT: Z = 1.707, *p* = 0.088; SR: Z = 1.406, *p* = 0.160) did not change significantly between pre- and post-treatment assessments.

### 3.3. Lung Function Tests and Association with Perfusion Defects

**Figure 4:**
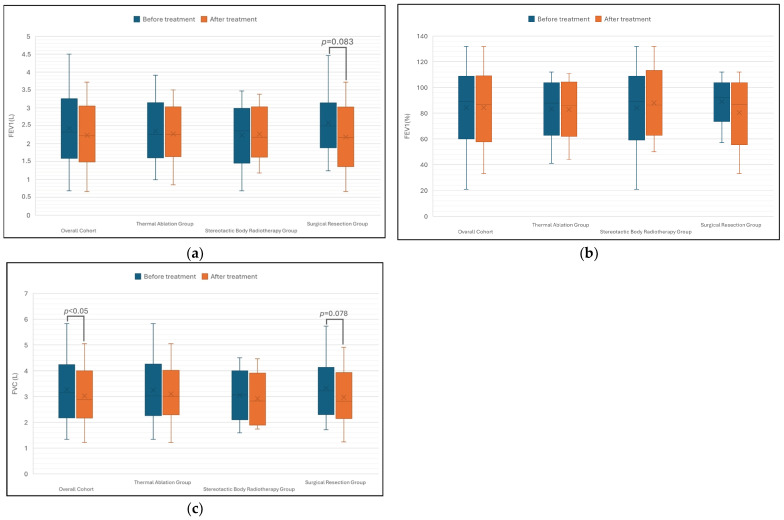
Changes in pulmonary function parameters before and after treatment across different treatment groups (*n* = 45). (**a**) Forced expiratory volume in 1 s (FEV1) in liters (L); (**b**) forced expiratory volume in 1 s (FEV1) as a percentage of predicted value (%); (**c**) forced vital capacity (FVC) in liters (L). In each boxplot, the cross (×) indicates the mean and the horizontal line inside the box indicates the median. Statistical significance was defined as *p* < 0.05.

As illustrated in Figure 4, no statistically significant changes in FEV1 (L) or FEV1 (%) were observed in the overall cohort (*n* = 45) at follow-up (*p* = 0.063 and *p* = 0.142, respectively). Similarly, FEV1 did not change significantly within the individual SBRT, TA, and SR subgroups (*p* > 0.05). However, a decrease in median FEV1 was noted in the SR group (*p* = 0.083); although this result did not reach statistical significance, the effect was in the expected direction.

Regarding FVC, a significant reduction was observed from baseline to follow-up in patients across all treatment groups combined (*p* = 0.022). However, no significant changes were found when analyzing the groups separately (*p* > 0.05). Nevertheless, the surgery group demonstrated a modest trend towards significance regarding FVC reduction (*p* = 0.078).

Pulmonary function differed significantly according to the severity of perfusion defects on scintigraphy both before and after treatment. Significant group differences were observed for FEV_1_% (H (4) = 14.28, *p* = 0.006 pre-treatment; H (4) = 12.76, *p* = 0.013 post-treatment) and FVC% (H (4) = 10.31, *p* = 0.035; H (4) = 9.68, *p* = 0.046). Patients without perfusion defects showed the highest mean ranks for FEV_1_% (42.3 pre-treatment; 31.6 post-treatment) and FVC% (37.9; 31.8), whereas those with more extensive or residual defects had progressively lower values (lowest mean ranks 16.6 and 19.9 for FEV_1_% and FVC%, respectively), indicating a stepwise decline in lung function with increasing defect severity. No significant differences were observed for FEV_1_ (L) (*p* = 0.208 pre; *p* = 0.086 post), FVC (L) (*p* = 0.502; *p* = 0.335), or DLCOc (mmol/min/kPa) (*p* = 0.442; *p* = 0.120), while DLCOc% (*p* = 0.266; *p* = 0.05) approached statistical significance post-treatment, suggesting a trend toward reduced diffusing capacity in patients with more extensive perfusion abnormalities.

**Figure 5:**
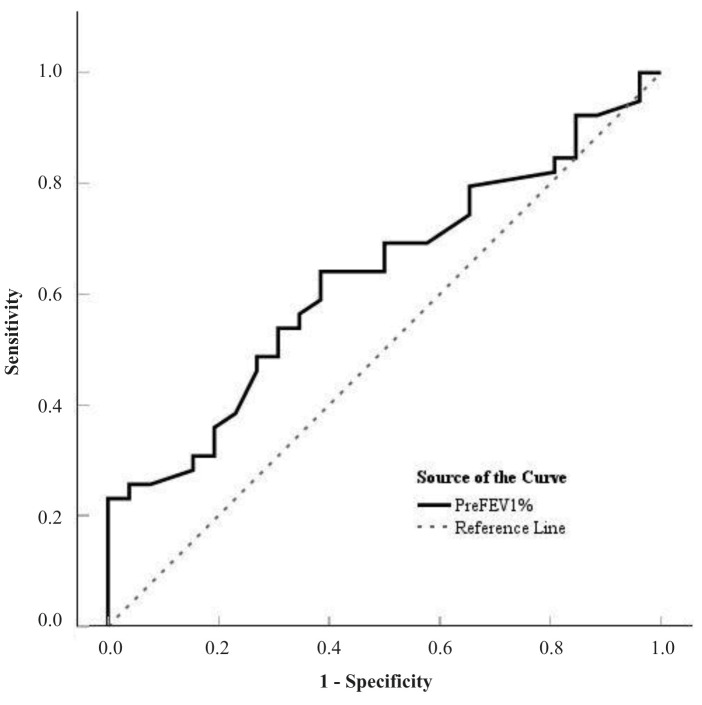
Predictive value of pre-treatment (FEV1%) for identifying significant pulmonary perfusion defects (*n* = 45). The area under the curve (AUC) was 0.629 (95% CI: 0.494–0.764, *p* = 0.062). An optimal threshold of 94% yielded a sensitivity of 64.1% and a specificity of 61.5%. Abbreviation: FEV1, forced expiratory volume in 1 s.

Pre-treatment spirometry parameters showed a limited ability to identify patients with perfusion defects on scintigraphy (Figure 5). The strongest association was found for FEV_1_% (AUC = 0.629, 95% CI 0.494–0.764, *p* = 0.062), while FVC% showed a similar tendency (AUC = 0.603, 95% CI 0.467–0.739, *p* = 0.074). Although these findings were not statistically significant, a slight trend toward lower spirometry values in patients with perfusion defects could be observed. The optimal threshold for FEV_1_% was identified as 94%, corresponding to a sensitivity of 64.1% and a specificity of 61.5%. When this cut-off value was applied, patients with FEV_1_% below 94% were more likely to have perfusion defects (χ^2^ (1) = 4.13, *p* = 0.042; OR = 2.86, 95% CI 1.02–7.97).

**Figure 6:**
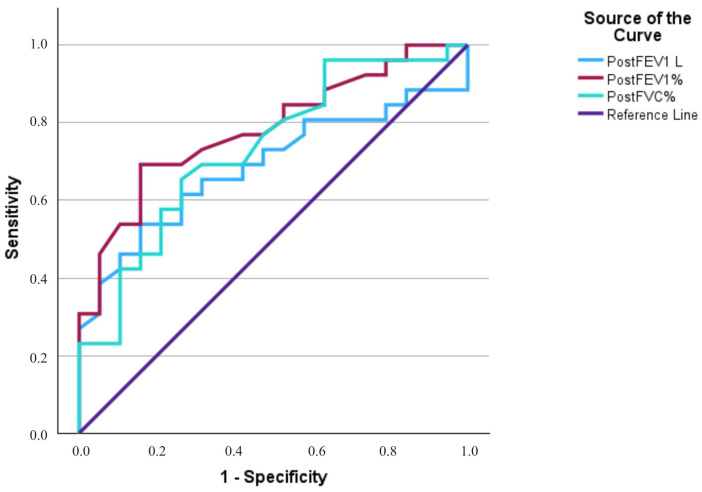
Diagnostic accuracy of post-treatment spirometry parameters for predicting pulmonary perfusion defects (*n* = 45). The ROC curves represent FEV1% (AUC = 0.783; 95% CI: 0.651–0.916; *p* < 0.001), FVC% (AUC = 0.733; 95% CI: 0.585–0.881; *p* = 0.002), and FEV1 (L) (AUC = 0.687; 95% CI: 0.532–0.843; *p* = 0.018). FEV1% demonstrated the highest diagnostic accuracy. Abbreviations: FEV1, forced expiratory volume in 1 s; FVC, forced vital capacity.

After treatment, spirometry parameters showed a markedly stronger association with residual perfusion defects. The strongest association was found (Figure 6) for FEV_1_% (AUC = 0.783, 95% CI 0.651–0.916, *p* < 0.001), followed by FVC% (AUC = 0.733, 95% CI 0.585–0.881, *p* = 0.002) and FEV_1_ (AUC = 0.687, 95% CI 0.532–0.843, *p* = 0.018). The optimal thresholds identified were 2.10 L for FEV_1_ (sensitivity 53.8%, specificity 84.2%), 83.5% for FEV_1_% (sensitivity 69.2%, specificity 94.2%), and 91% for FVC% (sensitivity 65.4%, specificity 73.7%). When these cut-off values were applied, all these parameters demonstrated significant associations with the presence of post-treatment perfusion defects. Patients with FEV_1_ below 2.10 L were significantly more likely to have residual perfusion abnormalities (χ^2^ (1) = 6.76, *p* = 0.009; OR = 6.22, 95% CI 1.45–26.64). Similarly, lower FEV_1_% (<83.5%) and FVC% (<91%) were associated with residual perfusion abnormalities (χ^2^ (1) = 12.60, *p* < 0.001; OR = 12.00, 95% CI 2.71–53.14, and χ^2^ (1) = 6.71, *p* = 0.010; OR = 5.29, 95% CI 1.44–19.45, respectively). These results indicate that reductions in post-treatment FEV_1_, FEV_1_%, and FVC%—particularly below these cut-off values—serve as reliable predictors of persistent perfusion impairment on scintigraphy.

## 4. Discussion

Most commonly, surgical resection involves lobectomy, with an approximate volume of 1000–1500 mL for a single lobe [9,10,11,12]. After thermal ablation, changes in perfusion function and lung volume on SPECT/CT are usually minimal, as the ablated areas are small (typically <50 mL) and perfusion loss remains localized [13,14,15]. Our study showed slightly more variation, but the overall tendency was similar. Thermal ablation and radiotherapy were associated with more limited changes in lung perfusion on SPECT/CT, indicating that both modalities are generally more lung-perfusion-sparing compared with surgical resection. These findings are consistent with recent evidence suggesting that CT—guided microwave ablation causes only limited and reversible pulmonary function impairment, further supporting its role as a lung-parenchyma-sparing therapy [16]. Our results reinforce the notion that thermal ablation may be particularly suitable for patients with limited pulmonary reserve, as it minimizes the loss of functional lung tissue compared to more invasive surgical options. It is important to note that the follow-up period in this study was limited to three months post-treatment. While this timeframe allows for the assessment of acute changes, it may not capture the full extent of lung injury or remodeling. For instance, radiation-induced lung injury and perfusion changes often evolve over 6 to 12 months, and the transition from inflammatory responses to stable fibrosis following thermal ablation can also take longer than 90 days. Therefore, our findings should be framed strictly as early post-treatment functional assessments, and caution should be exercised when extrapolating these data to long-term clinical outcomes.

Although changes in lung function across the different treatment groups did not reach statistical significance, patients who underwent lung resection demonstrated a modest trend toward reduced FEV_1_ and FVC. This lack of statistical significance is likely related to the limited sample size in our cohort. Notably, previous studies have shown that patients undergoing surgical treatment typically experience greater declines in pulmonary function compared with those treated with thermal ablation or radiotherapy [17,18]. Consistent with previously published results regarding thermal ablation, the data from our study support that thermal ablation is a lung-function-sparing treatment [16,19]. This supports that, similar to radiotherapy, thermal ablation can be safely used in patients who are not eligible for surgical treatment.

Spirometry correlates moderately to strongly with lung perfusion defects detected via lung perfusion SPECT/CT, but cannot reliably predict all regional perfusion abnormalities, as SPECT/CT provides a more sensitive and detailed assessment of regional perfusion loss than spirometry alone. Recent studies in patients with interstitial lung disease and lung cancer have demonstrated that FEV_1_% and DLCOc % show significant correlations with SPECT/CT-derived perfusion scores and functional lung volumes (r = 0.58–0.80), particularly when semi-quantitative SPECT/CT thresholds are applied, although SPECT/CT can still detect regional perfusion defects not reflected in global spirometry values [20,21,22]. In our study, a significant association between FEV_1_% and perfusion defect severity was also observed, whereas no relationship with DLCOc was detected, most likely due to the relatively small sample size. Evaluation is further complicated by the fact that the EANM guidelines for lung perfusion scintigraphy are based solely on planar imaging rather than SPECT or SPECT/CT, and the recommended classification only indicates whether pulmonary embolism is likely or unlikely, without considering the number of perfusion defects or their severity [3].

Lung perfusion scintigraphy identifies perfusion defects that may be due to perfusion abnormalities suggestive of possible vascular impairment, or pulmonary diseases, which are not always apparent on CT imaging [23]. Recent studies demonstrate that SPECT/CT is more sensitive than planar scintigraphy and can alter management in marginal surgical candidates by detecting perfusion defects that impact predicted postoperative lung function, even when CT does not show corresponding anatomical abnormalities [24]. Additionally, perfusion scintigraphy is recommended for the detection and follow-up of thromboembolic disease, as it can identify perfusion defects from chronic thromboembolic pulmonary hypertension or subclinical embolism that may be missed on routine imaging [25]. Incidental perfusion abnormalities suggestive of possible vascular impairment are particularly common, accounting for up to two-thirds of all cases reported in the literature [26,27,28]. This observation is consistent with our findings, as perfusion defects—which could not be explained by causes other than possible vascular impairment—were identified in more than half of the patients in our cohort before treatment. Moreover, perfusion defect severity before treatment was significantly associated with its severity after treatment, suggesting consistency over time. The incidence of perfusion defects due to possible vascular impairment in these patients is elevated due to cancer-related hypercoagulability, patient-specific factors such as advanced age and comorbidities, as well as treatment-related risks including surgical intervention, chemotherapy, and central venous catheterization [26,29,30,31]. Despite these findings, dedicated CT or SPECT/CT imaging specifically to screen for perfusion abnormalities is not routinely performed before selecting a lung cancer treatment strategy. Perfusion defects due to vascular impairment occur more frequently after surgical treatments for lung cancer, such as lobectomy or atypical resection, than after radiotherapy or thermal ablation. The highest risk is observed within the first three months postoperatively. Radiotherapy is associated with a moderate risk of perfusion abnormalities. It is important to evaluate these regions carefully, as radiation can cause a significant drop in overall lung function (such as FEV1) if it hits areas with high blood flow. By using SPECT/CT to identify these active zones, clinicians can plan radiation to avoid healthy tissue and reduce the risk of lung injury [32]. In contrast, thermal ablation carries a lower risk of perfusion abnormalities, with complications more commonly including pneumothorax or pleural effusion rather than thromboembolic events [33,34,35,36,37,38]. In our study, although lung perfusion findings did not dictate the treatment strategy—due to the absence of clinical symptoms of embolism—the majority of patients with perfusion defects were treated with thermal ablation or radiotherapy. Given the high overall rate of these pre-existing findings, they emphasize the importance of considering vascular status as a relevant factor when selecting the most appropriate treatment strategy for lung cancer patients. Furthermore, lung perfusion SPECT/CT should be strongly considered for integration into routine pre-treatment assessment. Overall, while spirometry remains a valuable global measure of lung function, it cannot replace SPECT/CT for the identification and quantification of regional perfusion defects.

Our findings regarding baseline perfusion must be interpreted within the context of clinical selection criteria. We observed a significantly higher prevalence of baseline perfusion defects in the thermal ablation (80%) and SBRT groups compared to the surgical resection group (31.6%). This distribution likely reflects an inherent selection bias common in clinical practice: patients referred to for non-surgical, lung-sparing interventions often present with more advanced age, higher comorbidity profiles, or borderline pulmonary function that precludes them from radical surgery. Such patients are inherently more predisposed to chronic microvascular changes or previous thromboembolic events, manifesting as pre-existing perfusion impairment. While including these high-risk cohorts provides a more representative “real-world” view of the population undergoing lung cancer treatments, this non-randomized allocation may confound the comparison of post-treatment functional decline. Furthermore, our study faced a notable attrition rate for longitudinal follow-up. While SPECT/CT data remained complete for the entire cohort (*n* = 68), follow-up lung function testing (spirometry and DLCOc) was available for only 45 patients. This loss to follow-up, primarily due to patients’ inability to attend the 3-month hospital visit, may have limited the statistical power to detect smaller functional changes within the treatment subgroups. Consequently, while the perfusion findings are robust across the full cohort, the spirometric results should be interpreted with caution, acknowledging the potential for attrition bias. Lastly, a technical limitation of our study is the absence of ventilation imaging or CT pulmonary angiography. Therefore, the observed perfusion defects, while suggestive of potential vascular impairment or subclinical events, cannot be definitively classified as pulmonary embolism. These findings should be interpreted as regional perfusion changes secondary to treatment or pre-existing conditions, rather than confirmed embolic events. Future studies with larger, multicenter cohorts are warranted to validate these findings and further investigate the clinical impact of pre-treatment perfusion abnormalities on long-term patient outcomes.

## 5. Conclusions

Radiotherapy and thermal ablation appear to preserve lung function, whereas surgical resection may lead to greater functional loss, aligning with previously published evidence. The high proportion of patients presenting with perfusion abnormalities suggestive of possible vascular impairment before treatment highlights the importance of incorporating lung perfusion scintigraphy into pre-treatment evaluation, particularly when such impairments are suspected. Studies with larger cohorts are needed to support the findings of this study.

## Figures and Tables

**Table 1:** Scoring System for Pulmonary Perfusion Defects on SPECT/CT.

Score	Description/Modified Criteria	Study Interpretation	2019 EANM V/P SPECT/CT Interpretation
**0**	No perfusion defects	Without perfusion abnormalities	Non-PE
**1**	Only diffuse perfusion abnormalities or ≤1 subsegmental perfusion defect	Minimal perfusion abnormalities	Non-PE
**2**	One segmental or two subsegmental perfusion defects	Low-grade perfusion impairment	PE
**3**	One segmental and >2 subsegmental perfusion defects, or 3 subsegmental perfusion defects, possibly with diffuse perfusion abnormalities but still does not fit into the 4-th score.	Moderate-grade perfusion impairment	PE
**4**	Two or more segmental, or ≥4 subsegmental perfusion defects, with or without additional diffuse abnormalities.	High-grade perfusion impairment	PE

Abbreviations: EANM, European Association of Nuclear Medicine; V/P, ventilation/perfusion; SPECT/CT, single-photon emission computed tomography/computed tomography; PE, pulmonary embolism.

**Table 2:** Baseline characteristics of the patients and their treated lesions.

	Surgery	SBRT	Thermal Ablation	Total	*p*-Value
Age, mean ± SD, years	67.8 ± 7.9	72.5 ± 9.5	69.2 ± 8.5	70.2 ± 8.9	0.172
Smoking history, mean ± SD, years	33.5 ± 15.7	41.4 ± 10.2	31.3 ± 16.9	36.3 ± 14.3	0.165
Pack-years, mean ± SD, years	28.5 ± 17.9	40.1 ± 23.9	28.6 ± 16.7	33.4 ± 20.7	0.242
ECOG, number (%)					
0	8 (42.1%)	6 (20.7%)	8 (40.0%)	22	0.092
1	11 (57.9%)	19 (65.5%)	12 (60.0%)	42	
2	0	4 (13.8%)	0	4	
Lesion histology, number	19	33	25	77	
Primary, (%)	18 (94.7%) ^a^	25 (75.8%) ^a^	12 (48.0%) ^b^	55	0.002 *
Metastatic, (%)	1 (5.3%) ^a^	8 (24.2%) ^a^	13 (52.0) ^b^	22	

Different superscript letters (e.g., a, b) indicate significant differences between groups; identical letters (e.g., a, b) indicate no difference. Further details regarding specific histological subtypes (e.g., adenocarcinoma, squamous cell carcinoma) are provided in a separate analysis [7]. Abbreviations: SBRT, stereotactic body radiotherapy; ECOG, Eastern Cooperative Oncology Group; SD, standard deviation. * Statistical significance was defined as *p* < 0.05.

**Table 3:** Comparison of lung perfusion and volume changes: surgical resection versus thermal ablation and stereotactic body radiotherapy.

Parameter	Surgical Resection vs. Thermal Ablation	Surgical Resection vs. Stereotactic Body Radiotherapy
**Affected lung perfusion function (%)**	*p* = 0.004 (r = 0.51) ↓	*p* = 0.002 (r = 0.48) ↓
**Affected lung volume (mL)**	*p* = 0.001 (r = 0.45) ↓	*p* = 0.002 (r = 0.41) ↓
**Unaffected lung perfusion function (%)**	*p* = 0.005 (r = 0.38) ↑	*p* = 0.002 (r = 0.41) ↑
**Unaffected lung volume (mL)**	NS	NS

Arrows indicate direction of change in the resection group relative to comparator treatment. NS—not significant.

## Data Availability

The raw data supporting the conclusions of this article will be made available by the authors on request.

## Abbreviations

The following abbreviations are used in this manuscript:

AUC	Area Under the Curve
CI	Confidence Interval
CT	Computed Tomography
CZT	Cadmium Zinc Telluride
DLCOc	Diffusing Capacity of the Lungs for Carbon Monoxide (adjusted for hemoglobin)
EANM	European Association of Nuclear Medicine
ERS	European Respiratory Society
ESTS	European Society of Thoracic Surgeons
FEV1	Forced Expiratory Volume in 1 Second
FVC	Forced Vital Capacity
IQR	Interquartile Range
MAA	Macroaggregated Albumin
NSCLC	Non-Small Cell Lung Cancer
OR	Odds Ratio
OSEM	Ordered Subset Expectation Maximization
PE	Pulmonary embolism
PSFR	Point Spread Function Recovery
ROC	Receiver Operating Characteristic
SBRT	Stereotactic Body Radiotherapy
SD	Standard Deviation
SPECT	Single-Photon Emission Computed Tomography
SR	Surgical Resection
TA	Thermal Ablation
VOI	Volume of Interest
